# The Effects of Different Anesthesia Methods on the Treatment of Parkinson’s Disease by Bilateral Deep Brain Stimulation of the Subthalamic Nucleus

**DOI:** 10.3389/fnins.2022.917752

**Published:** 2022-05-26

**Authors:** Yue Lu, Lei Chang, Jinwen Li, Bei Luo, Wenwen Dong, Chang Qiu, Wenbin Zhang, Yifeng Ruan

**Affiliations:** ^1^Department of Functional Neurosurgery, The Affiliated Brain Hospital of Nanjing Medical University, Nanjing, China; ^2^Department of Anesthesiology, The Affiliated Brain Hospital of Nanjing Medical University, Nanjing, China

**Keywords:** deep brain stimulation, general anesthesia, local anesthesia, subthalamic nucleus, Parkinson’s disease, intraoperative microelectrode recording

## Abstract

**Background:**

Subthalamic nucleus deep brain stimulation (STN–DBS) surgery for Parkinson’s disease (PD) is routinely performed at medical centers worldwide. However, it is debated whether general anesthesia (GA) or traditional local anesthetic (LA) is superior.

**Purpose:**

This study aims to compare the effects of LA and GA operation methods on clinical improvement in patients with PD, such as motor and non-motor symptoms, after STN–DBS surgery at our center.

**Method:**

A total of 157 patients with PD were retrospectively identified as having undergone surgery under LA (*n* = 81) or GA (*n* = 76) states. In this study, the Unified Parkinson’s Disease Rating Scale Motor Score (UPDRS-III) in three states, levodopa-equivalent-daily-dose (LEDD), surgical duration, intraoperative microelectrode recording (iMER) signal length, postoperative intracranial volume, electrode implantation error, neuropsychological function, quality of life scores, and complication rates were collected and compared. All patients with PD were routinely followed up at 6, 12, 18, and 24 months postoperatively.

**Result:**

Overall improvement in UPDRS-III was demonstrated at postoperative follow-up, and there was no significant difference between the two groups in medication-off, stimulation-off state and medication-off, stimulation-on state. However, UPDRS-III scores in medication-on, stimulation-on state under GA was significantly lower than that in the LA group. During postoperative follow-up, LEDD in the LA group (6, 12, 18, and 24 months, postoperatively) was significantly lower than in the GA group. However, there were no significant differences at baseline or 1-month between the two groups. The GA group had a shorter surgical duration, lower intracranial volume, and longer iMER signal length than the LA group. However, there was no significant group difference in electrode implantation accuracy and complication rates. Additionally, the Hamilton Anxiety Scale (HAMA) was significantly lower in the GA group than the LA group at 1-month follow-up, but this difference disappeared at longer follow-up. Besides, there was no significant group difference in the 39-item Parkinson’s Disease Questionnaire (PDQ-39) scale scores.

**Conclusion:**

Although both groups showed overall motor function improvement without a significant postoperative difference, the GA group seemed superior in surgical duration, intracranial volume, and iMER signal length. As the accuracy of electrode implantation can be ensured by iMER monitoring, DBS with GA will become more widely accepted.

## Introduction

Parkinson’s disease (PD), the main pathological mechanism of loss of dopaminergic neurons in the substantia nigra, is one of the most common neurodegenerative diseases affecting the older adults, second only to Alzheimer’s disease (Tysnes and Storstein, 2017; Simon et al., 2020). Dopaminergic treatment can initially control the motor symptoms of PD; however, as the disease progresses, this treatment is likely to cause medication-related side effects, such as motor fluctuations, dyskinesia, and impulse control disorders (Heumann et al., 2014; Wang et al., 2021). Deep brain stimulation (DBS) surgery targeting the subthalamic nucleus (STN), which has become a mainstream therapy for advanced PD (Herrington et al., 2016), can effectively resolve the above side effects and reduce the required levodopa dosage after surgery (Krack et al., 2019). Furthermore, considering the effects of general anesthesia (GA) on intraoperative microelectrode recording (iMER), an awake technique is the standard approach for DBS electrode insertion at most centers (Tempelhoff, 2017; Bos et al., 2019; Vesper et al., 2022). During this process, intraoperative temporary stimulation is performed to verify the patient’s contralateral limb muscle tension, exercise ability, and any side effects and to determine the optimal electrode implantation target in the target nucleus (Kraus and Pohle, 2000; Chen et al., 2018a; Walker et al., 2019). However, some PD patients with severe medication-off symptoms and excessive anxiety cannot tolerate DBS placement in an awake state (Chen et al., 2018b; Asriyants et al., 2021). Consequently, it is preferable to perform DBS surgery while such patients with PD are asleep (Ko and Burchiel, 2018; Ko et al., 2018).

Although many centers have successfully performed DBS insertion under GA, few reports compare the outcomes of DBS surgery performed under GA or in an awake state with local anesthesia (LA). Here, we present postoperative clinical differences from multiple dimensions for patients with PD undergoing awake or asleep bilateral STN-DBS lead placement at a single center.

## Materials and Methods

### Patients and Data

A total of 157 patients with PD who underwent STN-DBS surgery at our center between January 2018 and January 2020. A multidisciplinary team performed the diagnosis and surgical indications of the patients based on the United Kingdom PD Brain Bank criteria and relevant expert consensus (Poewe et al., 2017). In addition to routine examinations to rule out surgical contraindications, each patient with PD underwent medication-on and medication-off Unified Parkinson’s Disease Rating Scale part III (UPDRS-III) testing preoperatively and showed an improvement rate of medication-on/off > 30%. Patients who demonstrated dementia or severe psychiatric disorders, a history of radiation-based therapy or DBS therapy for PD, or any other movement disorder were excluded. Patients with PD were not randomly assigned to groups; instead, awake or asleep DBS surgery was determined based on the inclination of the patients and evaluation by neurologists specializing in movement disorders. After surgery, all patients routinely visited the center for assessment of changes in motor function in the medication-off, stimulation-off state (OFF-OFF), the medication-off, stimulation-on state (OFF-ON), and the medication-on, stimulation-on state (ON-ON), and non-motor function.

### Neurosurgical Procedure

For the LA group, all dopaminergic medications except levodopa were discontinued at least 3 days before surgery, and levodopa was withdrawn at least 12 h preoperatively due to the intraoperative temporary stimulation test, which was not necessary for the GA group. The detailed surgical procedures were as follows: First, a stereotactic frame was installed under local infiltration anesthesia of the scalp. The two sides of the frame were placed parallel to the anterior commissure–posterior commissure (AC-PC) projection line between 2 cm above the lateral canthus and 3.5 cm above the outer ear hole. Next, a thin slice (1.0 mm) stereotactic computed tomography (CT) scan fused with a 3.0 T magnetic resonance imaging (MRI) scan, including sagittal T1 and axial T2/SWI sequences, was used to guide electrode implantation using the SurgiPlan system. Eventually, bilateral quadripolar electrodes were implanted into the STN under multimodal fusion imaging and intraoperative electrophysiology guidance. In the LA group, patients remained awake during electrode implantation, such as iMER and intraoperative test stimulation, which co-determined the final location of electrode implantation. In contrast, the GA group accepted endotracheal intubation after induction of anesthesia (fentanyl citrate 0.002–0.004 mg/kg, cis-atra library ammonium 0.15 mg/kg, and propofol 1.50–2.50 mg/kg) and the depth of anesthesia was monitored by the bispectral index (BIS) during maintenance of anesthesia (propofol 3–5 mg/kg⋅h and remifentanil 0.03–0.06 mg/kg⋅h). Propofol was completely stopped approximately 15–20 min before iMER, and remifentanil was reduced to 0.007 mg/kg⋅h to ensure a BIS value was 60 ± 5. After obtaining a satisfactory bilateral electrodeposition, the pulse generator was usually implanted into the subclavian subcutaneous fat layer on the patient’s left side under GA in both groups.

### Statistical Analysis

Statistical analysis was performed using SPSS Statistics v. 25 (IBM, United States). Continuous data are presented as the mean ± standard deviation (SD), while dichotomous data are expressed as percentages. A value of *p* < 0.05 was considered statistically significant. The independent samples *t*-test or the Mann–Whitney *U*-test depended on whether the data satisfied the normality criterion. A one-way repeated analysis of variance (ANOVA) was performed to compare UPDRS-III and LEDD after testing normality and homogeneity of variance. A Bonferroni correction was applied for multiple comparisons.

## Results

### Patient Characteristics

As shown in Table 1, the baseline characteristics were similar among patients with PD in the LA and GA groups.

**TABLE 1:** Baseline characteristics of patients in both the groups.

	LA group (*n* = 81)	GA group (*n* = 76)	*P*-value
Age(y)	63.7 ± 6.4	62.8 ± 6.2	0.346
Male	49 (60.5%)	46 (60.5%)	0.997
Female	32 (39.5%)	30 (39.5%)	0.997
Disease duration(y)	9.5 ± 2.9	9.3 ± 3.0	0.823
H and Y stage	3.29 ± 0.73	3.24 ± 0.66	0.841
UPDRS scores (Med OFF)	71.1 ± 16.8	67.5 ± 15.9	0.160
UPDRS-III scores (Med OFF)	38.8 ± 9.6	37.6 ± 9.3	0.660
UPDRS-III scores (Med ON)	20.1 ± 3.8	18.9 ± 4.8	0.059
Levodopa daily dose (mg/d)	1080.58 ± 51.35	1081.26 ± 57.75	0.898
UPDRS-III improvement (%)	47.1 ± 6.9	49.5 ± 7.2	0.064
HAMA scores	6.6 ± 3.4	6.0 ± 3.8	0.188
PDQ-39 scores	63.4 ± 10.0	65.0 ± 9.0	0.135

*LA, local anesthetic; GA, general anesthesia; y, year; H and Y stage, Hoehn and Yahr stage; UPDRS, Unified Parkinson’s disease Rating Scales; UPDRS-III, Unified Parkinson’s disease Rating Scale Motor Scores; HAMA, Hamilton Anxiety scale; PDQ-39, the 39-item Parkinson’s Disease Questionnaire.*

### Motor Function Assessment

For all patients (LA = 81 and GA = 76), UPDRS-III scores in three states (OFF-OFF, OFF-ON, and ON-ON) were analyzed at 6, 12, 18, and 24 months postoperatively. Both groups showed improvement in motor function with stimulation-on, although motor function tended to decline with longer follow-up.

### OFF-OFF

**FIGURE 1:**
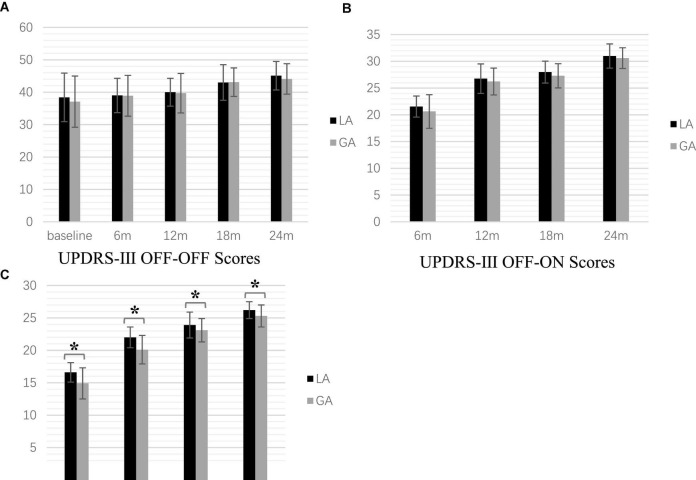
Postoperative motor symptom scale scores at the time point of follow-up (*P < 0.001). **(A)** UPDRS-β OFF-OFF Scores **(B)** UPDRS-β OFF-ON Scores **(C)**. UPDRS-β ON-ON Scores.

Significant differences were observed at the different time points within both group by one-way repeated measures ANOVA [LA: *F*(2.84, 227.11) = 66.91, *p* < 0.001, GA: *F*(2.41, 181.03) = 73.19, *p* < 0.001]. The *Post hoc analysis* with Bonferroni correction showed that the UPDRS-III scores increased gradually over time postoperatively for both groups. However, in the LA group, there were no significant differences between baseline and 6 months follow-up (*p* = 1.000), baseline and 12 months follow-up (*p* = 0.244), and 6 and 12 months follow-up (*p* = 0.568). In the GA group, there were no significant differences between 6 and 12 months follow-up or 18 and 24 months follow-up (*p* = 0.080 and *p* = 0.282, respectively). Two-way repeated measures ANOVA showed significant differences for follow-up time [*F*(1.40, 104.78) = 15.04, *p* < 0.001] but not group [*F*(1.00, 75.00) = 2.39, *p* = 0.126]. In addition, there was a significant interaction between follow-up time and group [*F*(2.09, 156.85) = 42.40, *p* < 0.001]. When the LA and GA groups were analyzed, there were no significant differences at baseline [*F*(1.00, 75.00) = 0.71, *p* = 0.404)] 6 months [*F*(1.00, 75.00) = 0.01, *p* = 0.930], 12 months [*F*(1.00, 75.00) = 0.05, *p* = 0.819], 18 months [*F*(1.00, 75.00) < 0.001, *p* = 1.000], and 24 months [*F*(1.00, 75.00) = 1.38, *p* = 0.244] (Figure 1A).

### OFF-ON

One-way repeated measures ANOVA revealed significant differences at different time points in both the LA and GA groups [LA: *F*(1.44, 115.28) = 269.18, *p* < 0.001, GA: *F*(1.41, 106.00) = 228.27, *p* < 0.001]. The *post hoc* analysis with Bonferroni correction indicated statistically significant increases at all postoperative follow-up times (all *p* < 0.001). Two-way repeated measures ANOVA showed significant differences for the follow-up time [*F*(2.13, 159.50) = 124.11, *p* < 0.001], but no significant group difference [*F*(1.00, 75.00) = 0.46, *p* = 0.499]. Furthermore, there was a significant association between follow-up time and group interaction [*F*(2.14, 160.48) = 260.95, *p* < 0.001]. However, there was no significant difference between the GA and LA groups at baseline [*F*(1.00, 75.00) = 0.71, *p* = 0.404], 6 months [*F*(1.00, 75.00) = 3.92, *p* = 0.051], 12 months [*F*(1.00, 75.00) = 3.14, *p* = 0.081], 18 months [*F*(1.00, 75.00) = 3.22, *p* = 0.077], and 24 months [*F*(1.00, 75.00) = 2.61, *p* = 0.110] (Figure 1B).

### ON-ON

[Frame6]Two-way repeated measures ANOVA showed significant differences for follow-up time [*F*(1.00, 75.00) = 12.66, *p* = 0.001] and group [*F*(1.45, 108.60) = 916.36, *P* < 0.001], but not the interaction [*F*(1.30, 97.38) = 0.79, *p* = 0.407]. One-way repeated measures ANOVA revealed significant differences at 6 months [*F*(1.00, 75.00) = 31.89, *p* < 0.001], 12 months [*F*(1.00, 75.00) = 41.93, *p* < 0.001], 18 months [*F*(1.00, 75.00) = 5.03, *p* = 0.028], 24 months [*F*(1.00, 75.00) = 14.50, *p* < 0.001], but not the baseline [*F*(1.00, 75.00) = 0.71, *p* = 0.404], among the two groups (Figure 1C).

### Levodopa Equivalent of Daily Dose Reduction

**FIGURE 2:**
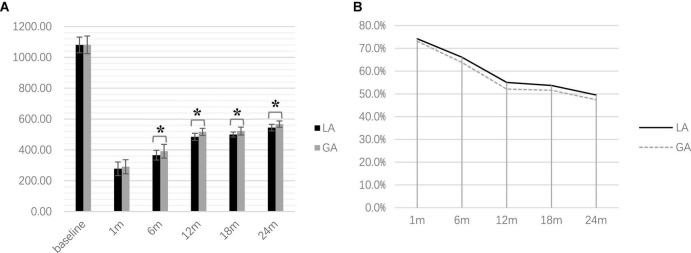
Postoperative levodopa-equivalent-daily-dose at the time point of follow-up (*P < 0.001). **(A)** Comparison of LEDD between two groups **(B)** LEDD reduction during postoperative follow-up.

One-way repeated measures ANOVA showed a significant difference in LEDD with follow-up time for both groups [LA: *F*(3.71, 296.38) = 3,975.92, *p* < 0.001, GA: *F*(3.29, 246.59) = 4,064.22, *p* < 0.001]. The *post hoc* analysis with Bonferroni correction showed a statistically significant reduction in both groups compared with the preoperative baseline (both *p* < 0.001). Mann–Whitney *U*-tests revealed that LEDD in the LA group (6, 12, 18, and 24 months postoperatively) were significantly lower than those in the GA group (all *p* < 0.001); however, there were no significant differences at baseline or 1 month between the two groups (*p* = 0.898 and *p* = 0.058, respectively) (Figure 2).

### Surgical Duration and Intraoperative Microelectrode Recording Signal Length

The two-sample *t*-test showed that the surgical duration (lead placement only) in the GA group was significantly shorter than that in the LA group (*p* < 0.001; LA: 179.7 ± 6.3 min; GA: 109.8 ± 7.3 min). In contrast, bilateral iMER signal length in the GA group was significantly longer than in the LA group (Table 2).

**TABLE 2:** Bilateral intraoperative microelectrode recording (iMER) signal length comparison.

	LA group (*n* = 81)	GA group (*n* = 76)	*P*-value
Left (mm)	5.4 ± 0.7	5.8 ± 0.8	<0.001
Right (mm)	5.6 ± 0.6	5.9 ± 0.6	0.001

*LA, local anesthetic; GA, general anesthesia.*

### Postoperative Intracranial Volume and Electrode Implantation Error

**FIGURE 3:**
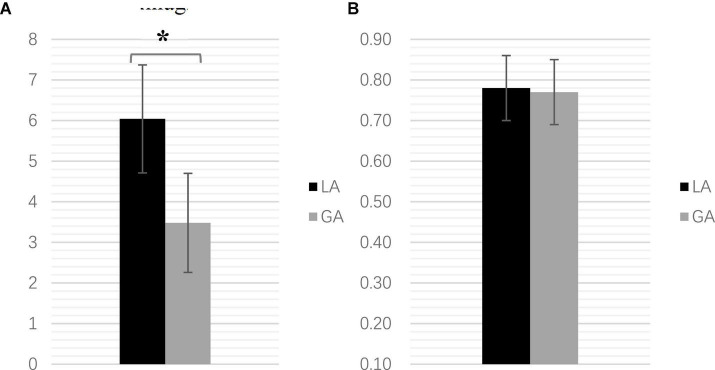
**(A)** The intracranial volume comparison (mL) (*P < 0.001). **(B)** The electrode implantation error comparison (mm).

On the first postoperative day, head CT showed a significant difference between the LA and GA groups in terms of intracranial gas accumulation (6.04 ± 1.33 vs. 3.48 ± 1.22 mL, *p* < 0.001) (Figure 3A). However, there was no significant difference in the accuracy of electrode implantation between the two groups (LA: 0.78 ± 0.08 mm; GA: 0.77 ± 0.08 mm, *p* = 0.606), as shown by the fused CT 1 week after surgery and preoperative imaging plans (Figure 3B).

### Hamilton Anxiety Scale and 39-Item Parkinson’s Disease Questionnaire

The Mann–Whitney *U*-tests showed that the postoperative Hamilton Anxiety **(**HAMA) (1 month) scores in the GA group were significantly lower than those in the LA group (*p* < 0.001). However, there were no significant group differences in HAMA scores at baseline or the other follow-up time points. Additionally, there were no significant group differences at baseline or postoperative follow-up in the 39-item Parkinson’s Disease Questionnaire (PDQ-39) scores (Table 3).

**TABLE 3:** Neuropsychological function and quality of life scale assessment.

	HAMA			PDQ-39		
	LA group (*n* = 81)	GA group (*n* = 76)	*P*-value	LA group (*n* = 81)	GA group (*n* = 76)	*P*-value
Baseline	6.6 ± 3.4	6.0 ± 3.8	0.188	64.0 ± 9.6	64.7 ± 7.8	0.135
1 month	8.4 ± 3.5	5.0 ± 3.3	< 0.001	–	–	–
6 month	5.2 ± 2.9	4.7 ± 3.0	0.227	43.0 ± 5.9	44.1 ± 7.5	0.410
12 month	5.1 ± 3.5	4.0 ± 3.2	0.056	40.6 ± 5.7	42.4 ± 9.1	0.202
18 month	5.2 ± 3.9	4.7 ± 2.5	0.923	43.6 ± 5.4	44.9 ± 8.2	0.550
24 month	5.4 ± 3.7	4.6 ± 2.5	0.392	48.0 ± 5.1	48.7 ± 7.8	0.761

*HAMA, Hamilton Anxiety scale; PDQ-39, the 39-item Parkinson’s Disease Questionnaire; LA, local anesthetic; GA, general anesthesia.*

### Complications

No intracranial hematoma, ischemic infarction, or intracranial infection occurred in either group after DBS lead placement. Delayed skin incision infection occurred in two cases in each group (one case each of retroauricular incision infection and frontal incision infection in the LA group; two cases of frontal skin incision infection in the GA group).

## Discussion

Since the advent of DBS surgery, lead placement in an awake state has become the standard procedure. The location and depth of electrode implantation are determined by iMER and temporary stimulation tests to minimize the adverse effects of stimulation (Blume et al., 2017; Mehanna et al., 2017; Walker et al., 2019; Frequin et al., 2020). However, lead placement under GA is a better option for some PD patients with severe off-medication symptoms and anxiety. In 2006, Hertel et al. (2006) first reported STN-DBS surgery under GA with intraoperative MER guidance. Many neurosurgery centers are starting to perform asleep electrode insertion using intraoperative imaging (e.g., CT and MRI) or MER guidance to ensure electrode implantation accuracy.

Our study showed that motor function improved postoperatively in the GA and LA groups, as assessed by UPDRS-III motor scores. Notably, UPDRS-III (ON-ON) improvement in the GA group was significantly greater than that in the LA group at postoperative follow-up. However, the group differences in UPDRS-III (OFF-OFF and OFF-ON) disappeared over long-term follow-up. Given these findings, there are two primary considerations. On the one hand, the surgery itself did not stop symptoms from worsening over time, and disease progression was generally similar between both groups. There was no significant difference between the groups in terms of just the postoperative efficacy of DBS stimulation-on in improving motor symptoms. On the other hand, it is possible that disease progression post-GA operation was faster compared with the post-LA operation, increasing the demand for drugs in the GA group. Due to greater PD drug use in the GA group postoperatively, the UPDRS-III (ON-ON) scores were significantly lower than those of the LA group due to the significantly lower LEDD after surgery in the LA group.

In terms of non-motor function, this study demonstrated significant differences in emotion during the short postoperative period, but the differences disappeared over long-term follow-up. However, the psychological status of the patients after DBS surgery was susceptible to various factors, such as the influence of the postoperative programming state. So the difference in the psychological scores could not be attributed to the impact of different anesthesia methods. Nevertheless, given that patients in the LA group remained awake during electrode implantation and had a longer surgical duration, this highlights the advantages of GA surgery, which can provide a more comfortable experience and broaden the patient population.

Our study found no significant differences in the incidence of intracerebral hemorrhages or infections between the LA and GA group. However, the awake group had a larger intracranial air volume (Blasberg et al., 2018; Ko et al., 2018; Jin et al., 2020). There are two possible explanations. First, the surgical duration in the LA group was longer. Second, the intraoperative uncontrollable cough and quiescent tremor in patients with PD increased cerebrospinal fluid loss, further conducive to increased intracranial volume.

The clinical prognosis of patients with PD after DBS implantation is closely related to electrode implantation accuracy (Chen et al., 2015; Li et al., 2016). Different anesthesia protocols involve different methods of ensuring that electrodes are implanted at the intended target (Li et al., 2021). Intraoperative verification of the actual targets depended on MER and the stimulation test in the LA group and MER or intraoperative imaging in the GA group. In our study, the electrophysiological signal length is longer under GA than under LA. The main reason for this result is that every patient in the GA group received microelectrode recording during microelectrode insertion. Most typical STN discharge waveforms can be recorded by selecting the ideal anesthesia protocol and monitoring the anesthesia depth (60 ± 5) through BIS (Jain et al., 2007; Kinfe and Vesper, 2013; Zeiler et al., 2013; Chakrabarti et al., 2014; Bezchlibnyk et al., 2020). However, during local anesthesia surgery, some patients suffer excessive nervousness, coughing, or severe tremors, which would interfere with electrophysiological signals. Since intraoperative images are not used at our center, mainly due to brain shifts caused by intraoperative cerebrospinal fluid loss, there are inevitable errors in the fusion of intraoperative images and preoperative planned images to determine the location of electrode implantation (Larson et al., 2018). However, iMER can monitor whether the microelectrode has entered the target nucleus and the depth of insertion, which has become the gold standard for accurate DBS surgery, requiring no intraoperative imaging. Finally, it should be noted that this study is subjected to limitations in terms of selection bias since patients with PD were not randomized to the GA or LA groups.

## Conclusion

Although the implantation of electrodes under LA is the standard DBS procedure, GA is becoming increasingly common due to improvements in anesthesia protocols and technology. Our study found no significant differences between GA and LA in improving motor symptoms or postoperative complication incidence in patients with PD undergoing DBS surgery. However, GA might be superior to LA in terms of short-term postoperative changes in non-motor symptoms. The possible advantages of DBS surgery under GA are that it is more acceptable and suitable for more patients with PD, especially those with obvious motor symptoms and anxiety (Venkatraghavan and Sheshadri, 2017). However, due to the limitations of this study, we cannot conclude that DBS surgery under GA is better than LA. More randomized controlled trials are required to determine which method is ideal.

## Data Availability Statement

The original contributions presented in the study are included in the article/supplementary material, further inquiries can be directed to the corresponding author/s.

## Ethics Statement

The studies involving human participants were reviewed and approved by the Ethics Committee of the Brain Hospital affiliated with Nanjing Medical University. The patients/participants provided their written informed consent to participate in this study. Written informed consent was obtained from the individual(s) for the publication of any potentially identifiable images or data included in this article.

## Author Contributions

YL and LC designed and wrote the manuscript. CQ and BL were responsible for data collection. JL, WD, and YL were responsible for data processing and analysis. WZ and YR contributed to the design of the study. WZ edited and revised the manuscript. All authors contributed to and approved the final manuscript.

## Conflict of Interest

The authors declare that the research was conducted in the absence of any commercial or financial relationships that could be construed as a potential conflict of interest.

## Publisher’s Note

All claims expressed in this article are solely those of the authors and do not necessarily represent those of their affiliated organizations, or those of the publisher, the editors and the reviewers. Any product that may be evaluated in this article, or claim that may be made by its manufacturer, is not guaranteed or endorsed by the publisher.
